# Anthocyanin Accumulation in Muscadine Berry Skins Is Influenced by the Expression of the MYB Transcription Factors, *MybA1*, and *MYBCS1*

**DOI:** 10.3390/antiox5040035

**Published:** 2016-10-12

**Authors:** Lillian Oglesby, Anthony Ananga, James Obuya, Joel Ochieng, Ernst Cebert, Violeta Tsolova

**Affiliations:** 1 Center for Viticulture and Small Fruit Research, College of Agriculture and Food Science, Florida A & M University, 6505 Mahan Drive, Tallahassee, FL 32317, USA; lillian.oglesby@gmail.com (L.O.); jamesobuya@yahoo.com (J.O.); violeta.tsolova@famu.edu (V.T.); 2 Food Science Program, College of Agriculture and Food Sciences, Florida A & M University, Tallahassee, FL 32307, USA; 3 Faculties of Agriculture and Veterinary Medicine, University of Nairobi, P.O. Box 29053, Nairobi 00625, Kenya; jochieng@uonbi.ac.ke; 4 Department of Biological and Environmental Sciences, Alabama A & M University, 4900 Meridian Street, Normal, AL 35762, USA; ecebert@gmail.com

**Keywords:** anthocyanins, antioxidants, MYB gene, pigments, muscadine grapes, *MybA1*, *MYBCS1*

## Abstract

The skin color of grape berry is very important in the wine industry. The red color results from the synthesis and accumulation of anthocyanins, which is regulated by transcription factors belonging to the MYB family. The transcription factors that activate the anthocyanin biosynthetic genes have been isolated in model plants. However, the genetic basis of color variation is species-specific and its understanding is relevant in many crop species. This study reports the isolation of *MybA1*, and *MYBCS-1* genes from muscadine grapes for the first time. They are designated as *VrMybA1* (GenBank Accession No. KJ513437), and *VrMYBCS1* (*VrMYB5a*) (GenBank Accession No. KJ513438). The findings in this study indicate that, the deduced *VrMybA1* and *VrMYBCS1* protein structures share extensive sequence similarity with previously characterized plant MYBs, while phylogenetic analysis confirms that they are members of the plant MYB super-family. The expressions of *MybA1*, and *MYBCS1* (*VrMYB5a*) gene sequences were investigated by quantitative real-time PCR using in vitro cell cultures, and berry skin samples at different developmental stages. Results showed that *MybA1*, and *MYBCS1* genes were up-regulated in the veràison and physiologically mature red berry skins during fruit development, as well as in in vitro red cell cultures. This study also found that in ripening berries, the transcription of *VrMybA1*, and *VrMYBCS1* in the berry skin was positively correlated with anthocyanin accumulation. Therefore, the upregulation of *VrMybA1*, and *VrMYBCS1* results in the accumulation and regulation of anthocyanin biosynthesis in berry development of muscadine grapes. This work greatly enhances the understanding of anthocyanin biosynthesis in muscadine grapes and will facilitate future genetic modification of the antioxidants in *V. rotundifolia*.

## 1. Introduction

Muscadine grapes (*Vitis rotundifolia* Michx.) are considered important *Vitis* species because they contain several unique flavonoid compounds with beneficial nutraceutical properties [1,2,3]. They are the only grapes containing ellagic acid in the skin and possess high antioxidant levels in comparison to other fruits [4,5] and contain significantly higher concentration of anthocyanin and phenolic acid [6,7]. Muscadine grapes are also highly resistant to diseases, which enable viticulturists to grow them with minimal applications of pesticides in regions with high disease pressure [8,9].

Several studies have reported that, the ripening of muscadine berry involves several changes in cell wall composition that leads to fruit softening [10,11,12]. Deytieux et al. and Deytieux-Belleau et al. [13,14] considered these changes to be key phases in growth that can be used to determine the quality of both wine and table grapes, because they immediately precede harvesting. Grape berry is classified as a non-climacteric fruit based on its respiration rates, and several studies have suggested that abscisic acid may play a role in the ripening process, because its increase in concentration correlates with the ripening of berries [15,16,17]. Since the biosynthetic pathway of flavonoids in muscadine grapes, plays an important role in berry development and ripening, greater understanding of the genes that control such expression is essential. Therefore, investigation of structural and regulatory enzymes that control the flavonoid biosynthesis pathway in muscadine grapes needs to be studied.

During grape berry development, the onset of maturation begins at véraison, that is, the beginning of skin color-change in red and black cultivars when anthocyanin pigment accumulation starts in the skin cells and continues through the ripening phase. Hence, the characterization of skin tissue is an essential parameter for understanding grape ripening due to its key role in the development of compounds responsible for the quality of wine. Additionally, grape skins are of increasing interest because they are metabolically active during development and ripening, and may have an endocrinal function [18]. The accumulation of anthocyanin pigments in grape berry skin is an important determination of berry quality. Usually restricted to the skins of berries, these pigments provide essential cultivar differentiation for consumers and are implicated in the health benefits of grape berry. In grapes, pigment biosynthesis may be induced by light, particularly ultraviolet (UV) radiation, stress treatments, and enzymes [19,20,21]. Several studies have shown that anthocyanin biosynthetic enzymes are induced in coordination during the developmental process of grape berries [22,23,24,25]. In a study by Espley et al. [26], the authors suggested that, the expression of the genes encoding the biosynthetic enzymes is coordinately regulated by transcriptional regulatory proteins. Other studies have also reported that transcription factors (TFs) are involved in the regulation of genes in the anthocyanin biosynthetic pathway and the components of the regulatory complex controlling anthocyanin biosynthesis are conserved in higher plants [27]. According to Martin and Paz-Ares [28], MYB TFs have been shown to play an important role in transcriptional regulation of anthocyanins, indicating that plant MYBs control secondary metabolic pathways, plant development, and signal transduction [29]. The characteristics of these MYBs involve the structurally conserved DNA-binding domain consisting of single or multiple imperfect repeats and the ones associated with the anthocyanin pathway are of the two-repeat (R2R3) class [26].

The regulation of MYBs can also be specific to discrete subsets of structural genes acting early or late in the anthocyanin biosynthetic pathway [30]. In *Arabidopsis thaliana*, Stracke et al. [31] stated that, there are 126 R2R3 MYB TFs, which can be divided on the basis of their sequence into 24 subgroups. However, the R2R3 MYB factors that regulate anthocyanin biosynthesis have been shown to interact closely with basic helix-loop-helix (bHLH) TFs as reported by Mol et al. [32] and Winkel-Shirley [33]. MYB genes have also been studied in grapes [34,35,36] and determined to be involved in anthocyanin biosynthesis. Numerous studies [34,37,38,39], have determined that many of the white grape cultivars arose from multi-allelic mutations of the MYBA1 and MYBA2 genes, which control the final biosynthetic steps of anthocyanin synthesis. This is a glycosylation reaction mediated by the UDP-glucose flavonoid 3-*O*-glucosyltransferase (UFGT). Similarly, Deluc et al. [35], determined that MYB5a and MYB5B are involved in regulating several flavonoid biosynthesis steps. Six MYB transcription factors (MYBA1, MYBA2, MYB5a, MYB5b, MYBPA1, and MYBPA2) have been reported to be associated with the regulation of the structural genes in the flavonoid pathway [40]. Although studies of *MYB* genes as transcriptional regulators of the flavonoid pathway genes have been investigated in *V. vinifera* varieties, to date this has not been done in muscadine grapes. The control of anthocyanin accumulation in muscadine grapes is a key question in understanding and manipulating the color of the berries and in vitro cell lines. Identification of the factors that exert this control will provide tools for moderating the extent and distribution of anthocyanin-derived pigmentation in berries and cell tissues of muscadine grapes. In this study, we isolated, and cloned *MybA1*, and *MYBCS1* (*VrMYB5a*) from the berry skins of muscadine grapes for the first time. The gene sequences were then characterized using in silico analysis and eventually deposited in the GenBank. We also analyzed the transcriptional profile of these two genes in muscadine berry skin across three developmental stages using real-time PCR. We discovered a high coordination between transcription regulation of *VrMybA1*, and *VrMYBCS1* (*VrMYB5a*) with the accumulation profile of total anthocyanin in the development stages. The results from this study provide new knowledge on the characteristics of MYB genes in muscadine grapes.

## 2. Materials and Methods

### 2.1. Plant Materials, Growth Conditions, and Cell Culture Maintenance

Berry skins and red cell lines of muscadine grapes (“Noble” var.) were used in this study. Berries were harvested from the Florida A & M University vineyard at three different development stages (green, veraison, and physiological maturity). Veràison refers to the onset of ripening, while physiological maturity refers to berries with brix of 18 and above and ready for harvesting. Berries that were free of physical injuries and similar in size at every stage were chosen for mRNA extraction. They were washed with distilled water; the skins were peeled and immediately frozen with liquid nitrogen and stored at −80 °C until use. In vitro red cell cultures established from super-epidermal cells of red berries of muscadine grapes [41] were also used in this study. The cells were grown in a growth chamber at 23 °C under a white light (150 μE·m^−2^·s^−1^) with a 16 h light/8 h dark cycle. The developed callus produces anthocyanin that is red in color. Solid culture mediums were used to grow and maintain the cells. Grape cell cultures were maintained in B-5 media as previously described by reference [4,42]. The cells in solid media were sub-cultured every 30 days and cell suspensions were transferred to fresh liquid media every 12 days. To transfer the cells, light forceps were gently used to scoop off the top layers of the cells. They were gently spread onto new culture media in three medium size layers in each plate in order to give the cells enough room to multiply. In the liquid medium, approximately 2.5 mL of the cell suspension was transferred into a 25 mL Erlenmeyer flask with B-5 liquid medium and placed on a shaker at 135 rpm.

### 2.2. RNA Extraction, Gel Electrophoresis and cDNA Synthesis

Samples were prepared from the skins (green, veràison, and physiologically mature berries) as well as cell lines of “Noble” grape. Total RNA was isolated using the RNeasy Plant Mini Kit (Qiagen, Valencia, CA, USA) according to the manufacturer’s protocol. RNA was quantified using Nanodrop 3300 (Thermo Scientific, Swedesboro, NJ, USA), and the inactivity was inspected by formaldehyde agarose gel electrophoresis. Purified RNA was treated with RNase-free DNAse 1, and immediately frozen to −20 °C. Formaldehyde gel electrophoresis (1% agarose) was used to evaluate the RNA quality. The gel apparatus (including the gel tray and comb) was treated with RNase Away^TM^ (Molecular Bio-Products, Inc., San Diego, CA, USA) and rinsed with distilled water. Total RNA was used in primary gene expression profiling. The SuperScript First-strand Synthesis System for RT-PCR (Invitrogen, Carlsbad, CA, USA) was used to synthesize cDNA in a 20 mL reaction containing 1 mg of DNase I-treated total RNA, 20 mM Tris-HCl (pH 8.4), 50 mM KCl, 2.5 mM MgCl_2_, 10 mM dithiothreitol, 0.5 mg oligo (dT), 0.5 mM each of dATP, dGTP, dCTP, and dTTP, and 200U SuperScript II Reverse Transcriptase. RNA, dNTPs, and oligo (dT) were mixed first, heated to 65 °C for 5 min, and placed on ice until the addition of the remaining reaction components. The reaction was incubated at 50 °C for 50 min, and terminated by heat inactivation at 85 °C for 5 min. The cDNA product was treated with 1 µL of Rnase H (Invitrogen) for 20 min at 37 °C. An identical reaction without the reverse transcriptase was performed to verify the absence of genomic DNA (no-RT control). The cDNA was stored at −20 °C until it was ready for use.

### 2.3. Isolation of the VrMybA1, and VrMYBCS1 (VrMYB5a) Genes

To isolate *MybA1*, and *MYBCS1* cDNA clones from muscadine grapes, primers were designed based on the basis of conserved amino acid sequences of several published *MybA1* and *MYBCS1* sequences from other plants to amplify the CDS region. Based on the sequences of published genes and specific sequences of *V. vinifera MybA1* and *MYBCS1* (NCBI accession numbers AB097923, and AY555190), two fragments were amplified from cDNA by the primers *MybA1*-*F*: 5′-CACCATGGAGAGCTTAGGAGTTAGA-3′ and *MybA1*-*R*: 5′-GATCAAGTGATTTACTTGTGT-3′, for *VrMybA1* and primers MYBCS1-F: 5′-CACCATGAGAAATCCGGCATCTGC-3′ and MYBCS1-R: 5′-GGGAGACATGGAGTGTTTTTGA-3′, for *VrMYBCS1*. cDNA synthesized from mRNA of the veràison berry skins was used as a template. A high fidelity polymerase (Promega, Madison, WI, USA) was used for PCR using the following program: 95 °C for 5 min, then 35 cycles of 95 °C for 50 s, 55 °C for 50 s, and 72 °C for 90 s; followed by elongation at 72 °C for 10 min. The PCR products were separated by 1% agarose gel electrophoresis, and strong bands were clearly detected at 530 bp and 760 bp, respectively representing *MybA1* and *MYBCS1*. The agarose gel slice containing the DNA fragment of interest was purified by DNA gel extraction kit (Qiagen, Valencia, CA, USA) according to the operator’s manual.

### 2.4. Cloning and Sequencing of VrMybA1, and VrMYBCS1 (VrMYB5a) Genes

The PCR fragments of *VrMybA1*, and *VrMYBCS1* (*VrMYB5a*) genes were subsequently purified and cloned into pGEM-T Easy Vector (Promega). Vectors and PCR-amplified products were mixed and ligated overnight at 4 °C and transformed into *Escherichia coli* strain JM109. The putative recombinant plasmid-pGEM-*MybA1* and pGEM-*MYBCS1* were extracted for PCR analysis, and strong bands were clearly detected at 530 bp and 760 bp, respectively confirming the presence of the *MybA1* and *MYBCS1* inserts. The pGEM-*MybA1* and pGEM-MYBCS-1 plasmids were sequenced from both ends at Eurofins MWG/Operon (Huntsville, AL, USA). The sequences were compared with the genes in the GenBank database using BLAST program from the National Center for Biotechnology Information (NCBI), which indicated that the PCR product had 99% identity in the activity site with the reported *Vitis MybA1* and *MYBCS1*, which confirmed that the obtained genes are muscadine *MybA1* and *MYBCS1*.

### 2.5. Bioinformatics Analysis

The deduced amino acid sequence of *VrMybA1*, and *VrMYBCS-1* were aligned using the BioEdit Sequence Alignment Editor, version 5.0.9 (Department of Microbiology, North Carolina State University, Raleigh, NC, USA) [43]. Theoretical molecular weights (MW) and isoelectric points (pI) were calculated using the Compute pI/Mw tool (http://web.expasy.org/compute_pi/). Putative target localization of *VrMybA1*, and *VrMYBCS1* was predicted by using WoLF PSORT (http://psort.hgc.jp/form.html) [44]. The phylogenetic tree was constructed using an online program (http://www.phylogeny.fr/) [45]. The three-dimensional (3D) structure was built using the SWISS-MODEL program and illustrated with the PyMOL viewer.

### 2.6. Expression Pattern in Different Berry Stages and in Cell Cultures

The expression levels of *VrMybA1*, and *VrMYBCS1* genes at three stages of muscadine berry skin developments were determined by RT-PCR, using SYBR green method on a CFX96 real-time cycler (BIO-RAD, Hercules, CA, USA). Relative quantitative real-time PCR reactions were performed in a 96-well plate to monitor cDNA amplification, according to the manufacturer’s protocol. As a control, a parallel amplification reaction of *Actin* (a housekeeping gene) was performed. Each primer set was designed based on the 3′-end cDNA sequence of the corresponding gene. The specific primers used for RT-PCR were as follows: for *VrMybA1* 5′-GGAATAGATCCCAGAACCCAC-3′ (Forward), and 5′-TGGTTGCTTCCAATTTCTCCC-3′ (Reverse), giving a product of 140 bp and *VrMYBCS-1* 5′-GAGGTTGATCTCATGATTAGG-3′ (Forward), and 5′-TGGAACTGAACCTCCTTTTTG-3′ (Reverse), giving a product of 180 bp; for *Actin* 5′-TAGAAGCACTTCCTGTGGAC-3′ (Forward) and 5′-GGAAATCACTGCACTTGCTC-3′ (Reverse), giving a product of 120 bp. Each PCR reaction (20 μL) contained 0.6 μL primer F, R (10 μM), 1 μL cDNA (10 ng), and 10 μL SsoAdvanced^TM^ SYBR^®^ Green Supermix (Bio-Rad, Hercules, CA, USA). The RT-PCR conditions were: 1 cycle at 95 °C for 3 min; 35 cycles at 95 °C for 10 s and 60 °C (*VrMybA1*, *VrMYBCS-1*, and Actin) for 30 s, followed by a melt cycle from 65.0 °C to 95.0 °C. Three replicates of all RT-PCR reactions were carried out on each sample. Amplification efficiency of all primers used was primarily determined prior to sample investigation. Relative expression values were firstly calculated as 2^−ΔCT^, normalized against the internal control *Actin* gene. The maximal expression level of each gene observed served as a calibrator (1.0) respectively, and the rest was expressed as ratios in relation to the calibrator (relative expression ratio).

### 2.7. Analysis of Total Anthocyanin in the Berry Skins

Approximately 2 g of fresh tissue (callus and/or skins) were kept frozen at −80 °C, then homogenized with Bio Homogenizer (Biospec Products, Inc., Bartsville, OK, USA) at 5000 rpm for, 5 min, in 10 mL extraction solvent (Methanol: 1% HCl—1:1). It was then centrifuged for 15 min (4 °C) (Eppendorf 5804R, Swedesboro, NJ, USA) at 11,000 rpm. The supernatant was collected and the residue was homogenized and re-extracted two more times. The extract was combined and filtered through a 0.45 µm syringe filter and used for total anthocyanin assay.

The pH differential spectrophotometric method was used to measure total anthocyanin content [46]. Two portions of anthocyanin extracts were diluted (using pre-determined dilution factor) with potassium chloride buffer (0.025 M, pH 1.0) and sodium acetate buffer (0.4 M, pH 4.5), respectively. After 15 min, the absorbance of both dilutions was measured at 520 nm and 700 nm against water (Ultrospec 3100 Pro UV-Vis Spectrophotometer, GE Healthcare, Piscataway, NJ, USA). The corrected absorbance of the diluted sample and the monomeric anthocyanin pigment concentrations were calculated as described elsewhere [46]. The total monomeric anthocyanin concentration was expressed as mg cyanidin-3,5-diglucoside equivalents (molecular weight = 611.5254, molar absorptivity ɛ = 30175) per g dry weight (DW). Dry weights of muscadine berries skins and callus tissue were determined using MJ33 Compact Infrared Moisture Analyzer (Mettler Toledo, Greifensee, Switzerland).

## 3. Results

### 3.1. Isolation and Cloning of VrMybA1 and VrMYBCS1 Genes from Muscadine Berry Skin Tissues

**Figure 1:**
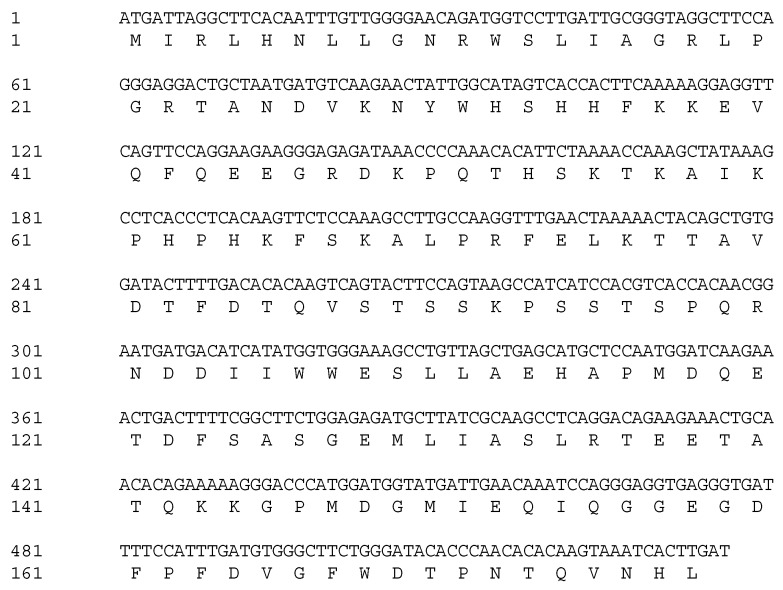
The complete cDNA sequence and amino acid sequence of the protein encoded by *MybA1* (GenBank accession number: KJ513437).

**Figure 2:**
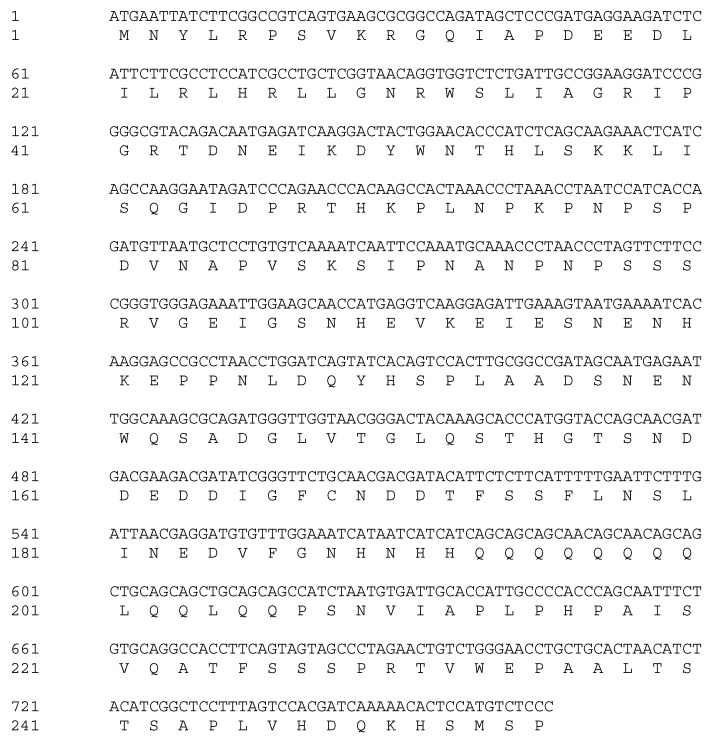
The complete cDNA sequence and amino acid sequence of the protein encoded by *MYBCS1* (GenBank accession number: KJ513438).

**Figure 3:**
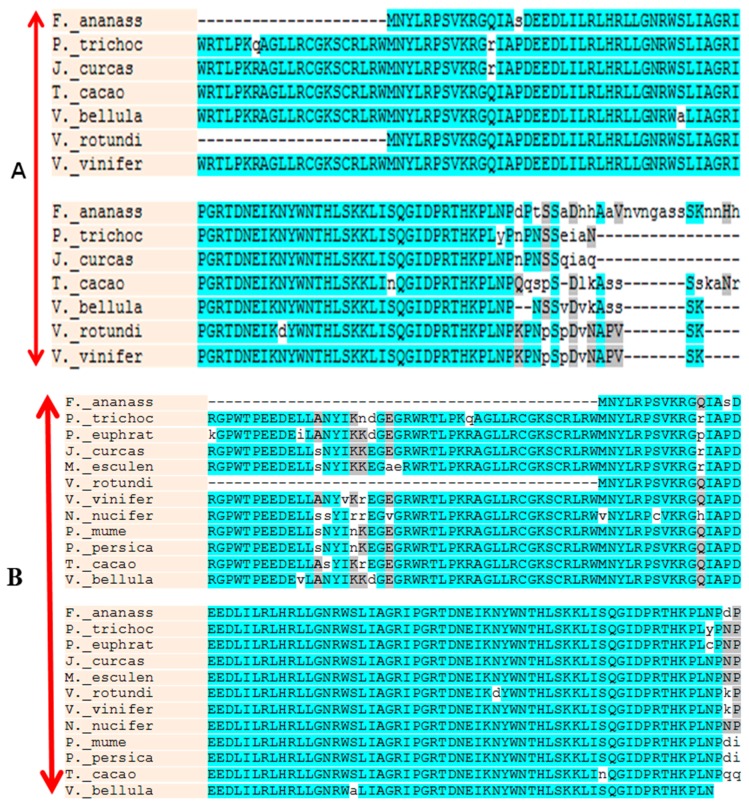
Amino acid sequence alignment of MybA1 (**A**); and MYBCS1 (**B**) proteins from different plant species. Residues highlighted in blue represent identical and similar amino acids, respectively. The alignment was performed using multi-align software as defined in the Materials and Methods section.

**Figure 4:**
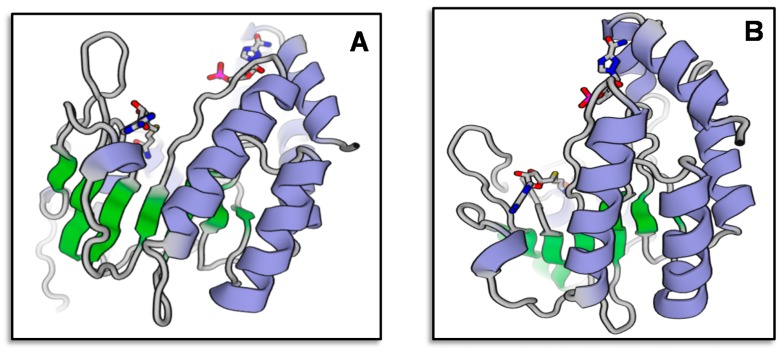
The computational modeled three dimensional structure of muscadine *MybA1* (**A**); and *MYBCS-1* (**B**).

Based on the alignment of homologous sequences from other plants, primer pairs were designed to amplify *VrMybA1* and *VrMYBCS1*. Using the homologous amplification method, these two regulatory genes were amplified successfully from the red grape skins. Agarose gel electrophoresis detection showed that the specific bands were 530 bp and 760 bp for *VrMybA1* and *VrMYBCS1*, respectively, which was consistent with the expected size based on the primer design. These bands were cloned and sequenced, and the exact sizes of *VrMybA1* and *VrMYBCS1* were determined to be 536 bp and 765 bp in length. After analysis and comparison of the sequences, we determined that the fragments had high similarity with known *MybA1* and *MYBCS1* genes from other plant species. The sizes obtained encoded protein sizes with 175 and 255 amino acid (AA) residues for VrMybA1 and VrMYBCS1, respectively (Figure 1 and Figure 2). The trimmed nucleotide sequences of *VrMybA1* and *VrMYBCS1* genes coding for proteins were submitted to the NCBI/GenBank database under the following accession numbers: *VrMybA1* (KJ513437) and *VrMYBCS1* (KJ513438). The results of homology BLAST showed that both MYB genes cloned in this study shared high sequence identity, ranging from 97% to 99%, with cDNA sequences from other species in the *Vitis* family in the NCBI/GenBank database. The deduced amino acid sequences showed high similarity (Figure 3A,B), and the three-dimensional (3D) structure of muscadine *VrMybA1* and *VrMYBCS1* (Figure 4) shared 90.3% similarity with the template. This further facilitated the positive identification of *VrMybA1* and *VrMYBCS1*.

### 3.2. Sequence Analysis of VrMybA1 and VrMYBCS1

Sequence analysis indicated that *VrMybA1* and *VrMYBCS1* were 536 bp and 765 bp in length with 121 and 253 bp on the 5′- and 3′-untranslated regions (UTRs), respectively for *VrMybA1* and 186 bp and 186 bp on the 5′- and 3′-UTRs, respectively for *VrMYBCS1*. The cDNA contained an open reading frame (ORF) of 536 bp for *VrMybA1* and 765 bp for *VrMYBCS-1* encoding 178 AA and 255 AA, respectively (Figure 1 and Figure 2). The deduced molecular mass (MW) of VrMybA1 protein was 20.2 kDA, with an isoelectric point (pI) of 6.3. For VrMYBCS-1 protein, the MW was 28.3 kDa with the pI of 5.65. SMART program (http://smart.embl-heidelberg.de/), was used to predict functional sites and results indicated there was a SANT domain, implicated in chromatin-remodeling and transcription regulation, between Trp85 and Phe135 for VrMybA1 (*E*-value 7.24 × 10^−2^) and for VrMYBCS-1 (*E*-value 2.29 × 10^−13^). The domain SANT/MYB of *VrMybA1* and *VrMYBCS-1* and their orthologues were compared with that of typical MYB domains found in plant MYB domain proteins, and with SANT domain often presented in proteins participating in response to anthocyanin accumulation. There was a broader search for genes with high homology to *VrMybA1* and *VrMYBCS-1* protein in other plant species and we found that *VrMybA1* and *VrMYBCS-1* contained a highly conserved DNA binding domain that was very similar to the DNA binding domains of other plant MYBs. The R2R3 imperfect repeats that bind to the target DNA sequences and are highly conserved among R2R3-MYB proteins were contained at the amino terminal. In *VrMYBCS-1*, there was a shorter sequence that is related to maize transcription factor activator C1 [47], and is needed to interact with a basic helix-loop-helix cofactor [35]. In addition to the C1 motif, a Gln-rich domain, QQQQQQQQLQQLQQP was identified. This motif resembles that found in *VvMYB5a* by reference [35]. Therefore, this study confirms that we have positively isolated and identified *VrMybA1* and *VrMYBCS-1* in muscadine grapes. This is very important in understanding the metabolic flux in the anthocyanin biosynthetic pathway of muscadine grapes.

**Figure 5:**
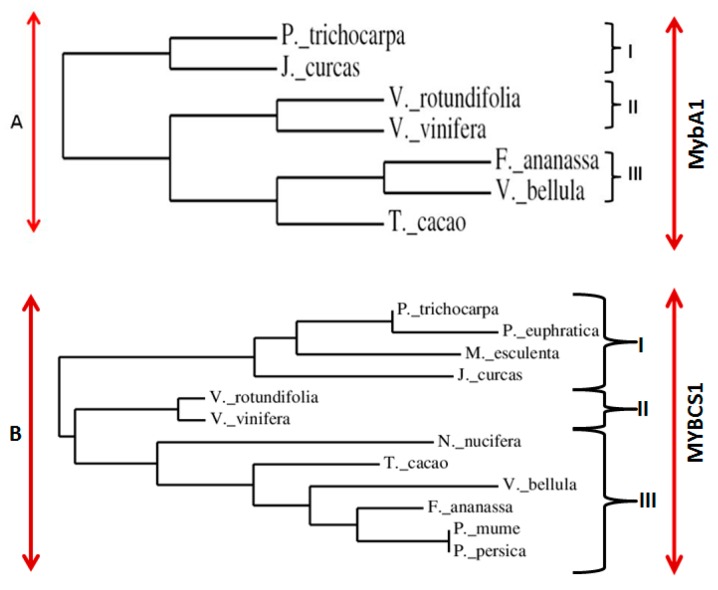
Comparison of the deduced amino acid sequences of R2R3-type MYB transcription factors from higher plants. (**A**) A phylogenetic tree for plant *MybA1* transcription factors; (**B**) A phylogenetic tree for plant *MYBCS1* transcription factors including the one isolated from muscadine grapes (*V. rotundifolia*) in the present study.

A phylogenetic tree based on amino acid alignment of *VrMybA1* and *VrMYBCS-1* and related proteins with SANT (Swi3, Ada2, N-Cor, and TFIIIB) and MYB domains was constructed using the neighbor-joining method of MEGA 4.0 [48]. The dendrograms indicated that VrMybA1 and VrMYBCS1 belong to a distinct cluster of MYB proteins from *Vitis* species (Figure 5A,B). MybA1 from different organisms were divided into three groups: I, II, and III (Figure 5A), and MYBCS1 was also divided into three groups: I, II, and III (Figure 5B). VrMybA1 from *V. rotundifolia* belonged to the subgroup II that is close to the *V. vinifera* and MYBCS1 from *V. rotundifolia* belonged to the subgroup II, which is also in the same clade as *V. vinifera*. Even though members of each subgroup have close relationships, the study of this relationship through phylogenetic analysis has enabled us to visualize how evolution has produced changes in the MYB gene family.

### 3.3. Expression of VrMybA1 and VrMYBCS1 Genes in Berry Skins Are Influenced by Physiological Changes

**Figure 6:**
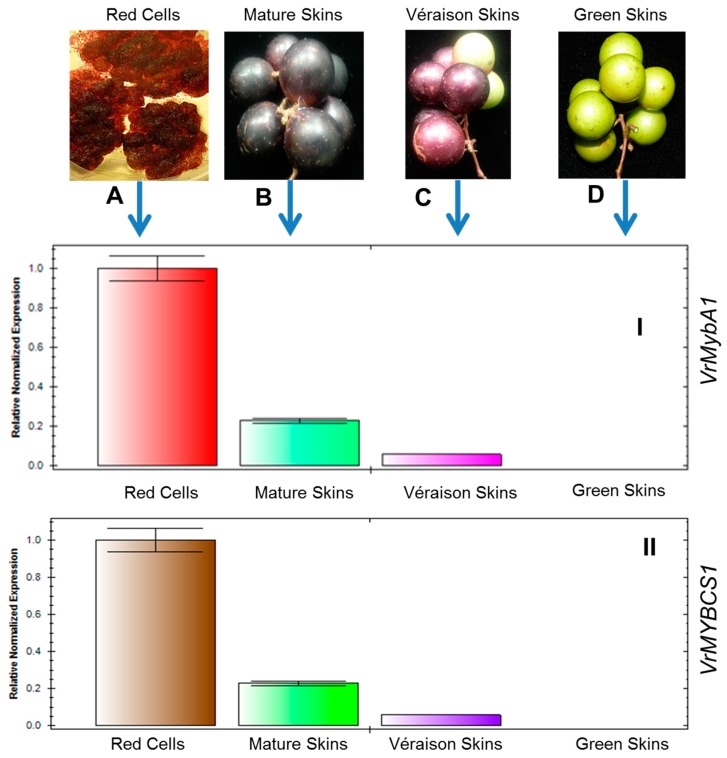
Plant samples used in the experiment (**A**) (Red cells); (**B**) (Mature berries); (**C**) (Veràison berries); and (**D**) (Green berries) and mRNA expression levels of *VrMybA1* (**I**); and *VrMYBCS-1* (**II**) genes in different developmental stages of muscadine grape berry skins. The values are expressed relative to a level of transcription (**I**) the veràison, which was set as 0.1. Values are the means of three replicates ± SE.

Real-time PCR was carried out to investigate the expression patterns of *VrMybA1* and *VrMYBCS1* genes induced by the physiological changes during grape berry developmental (Figure 6A–D). Real-time PCR analysis showed that the relative expression of *MybA1* (Figure 6I) and *MYBCS1* (Figure 6II) was significant among the tested samples. Expressions of these genes were not detected at the green stage. But at veràison, the skin color starts to change from green-to-red and the expressions of *VrMybA1* and *VrMYBCS-1* genes were already detectable in the red skin tissues. In the skins of physiologically mature berries, red pigments were actively developed, and levels of *VrMybA1* and *VrMYBCS-1* gene transcripts were greatly induced. To determine the contribution of *VrMybA1* and *VrMYBCS-1* in the flavonoid pathway of *V. rotundifolia*, the expression levels of these genes were analyzed in the in vitro cell cultures. The data obtained indicated that the expression levels of *MybA1* and *MYBCS-1* were significantly higher in the in vitro red cell lines as compared to that observed in the green, veràison, and mature skin tissues (Figure 6). Thus, this study confirms that *MybA1* and *MYBCS-1* play an important role in the accumulation of anthocyanin in *V. rotundifolia*.

### 3.4. Anthocyanin Accumulation in the Berry Skin and Cell Cultures

**Figure 7:**
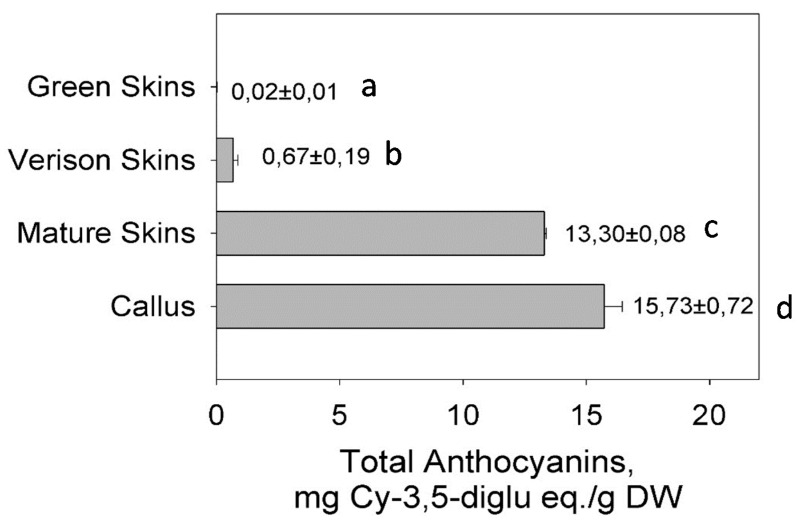
Analysis of anthocyanin accumulation in in vitro cell cultures (Callus), as well as in different development stages of muscadine berry skins (Mature, Veriason, and Green skins). Mean values with different small letters (**a**–**d**) are significantly different at (*p* = 0.01). The accumulation pattern of anthocyanins follows the pattern of expression of *VrMybA1*, and *VrMYBCS-1*.

Total anthocyanin was measured in the three phenotypes of berry skins (green, veràison, and physiologically mature). Total anthocyanin content was high in the in vitro red cell cultures and at a late stage of berry development or physiologically mature berry skins (Figure 7). The analysis indicated that anthocyanin accumulates progressively during berry development. It starts to accumulate on the berry skin at veràison and increases until it maximizes at physiological maturity. Comparatively, anthocyanin was low or non-existent at the green developmental stage (Figure 7). These results are consistent with the results shown in Figure 6I,II, where the red and green skin color differences were observed progressively in different stages of berry development. From these results, we can deduce that the accumulation of anthocyanin pigments in berry skin is likely influenced by the progressive expression of *VrMybA1* and *VrMYBCS-1*.

## 4. Discussion

MYB represent the largest number of proteins with active functions in numerous plant species. Thus far, 190 have been deduced in *Arabidopsis*, 156 in rice, and more than 200 have been identified in maize [49,50,51]. MYB transcriptional regulators contain conserved DNA-binding domains that are usually composed of one, two, or three imperfect 51 or 52 residue repeats (R1, R2, and R3). Each repeat encodes three α-helices, with the second and third helices forming a helix–turn–helix (HTH) structure when bound to DNA [52,53]. In plants, the overwhelming majority of MYB proteins are classified into a subfamily characterized by the presence of R2R3 motifs.

The genetics and biochemistry of anthocyanins and flavonoid biosynthesis in plant organ pigmentation are well established in model species. Although the molecular and biochemical characterization of *MybA1* and *MYBCS-1* or analogous genes in plants have been studied, most of the studies were carried out in model plant species, such as *Arabidopsis*, tobacco, and rice. However, *MybA1* and *MYBCS-1* or its analogues in muscadine grapes (*Vitis rotundifolia*), have not been reported before. The focus of this study was to investigate the existence and expression of *MybA1* and *MYBCS-1* in muscadine grapes and their possible involvement in anthocyanin biosynthesis. The cloning, characterization and expression of *MybA1* and *MYBCS-1* genes from muscadine grapes will create the possibility of elucidating the biosynthetic pathway in *V. rotundifolia*, which is now under intensive investigation in our laboratory.

Although regulatory genes as well as structural genes in flavonoid pathway have been isolated in other plant species, there are currently no reports of *VrMybA1* and *VrMYBCS-1* in *V. rotundifolia*. The results indicated that *VrMybA1* and *VrMYBCS*-1 are key enzymes in the anthocyanin-pigmentation pathway of *V. rotundifolia*, and the two genes are responsible for the formation of anthocyanin in the berry skins as well as the in vitro cell lines of *V. rotundifolia*. Flavonoids are responsible for many medical applications of grapes; therefore, the manipulation of the flavonoid biosynthetic pathway in muscadine grape cell cultures would provide an alternative to harvesting bioactive compounds from the grape cells [3,4]. *MybA1* and *MYBCS-1*, are key enzymes of the anthocyanin biosynthetic pathway, and can regulate the structural genes and catalyze the reaction(s) from the colorless leucoanthocyanidins to the colored anthocyanidins. The present research on isolation and characterization analysis of *MybA1* and *MYBCS-1* from muscadine grapes will provide new research opportunities for the overall metabolic flux control toward the targeted products using genetic engineering strategies.

## 5. Conclusions

In the present study, we successfully isolated and characterized two MYB genes (*VrMybA1* and *VrMYCS-1*) from muscadine grapes and analyzed their expression profiles in different stages of berry development as well as in in vitro cell lines for the first time. Phylogenetic relationship constructed based on the putative amino acid sequences demonstrated that *VrMybA1* and *VrMYCS-1* were most closely related to *V. vinifera*, among the surveyed plant species. Multiple alignments of amino acid sequences of *VrMybA1* and *VrMYCS-1* in relation to others showed that they have many active sites that are well-conserved in different plant species. Our data revealed that transcripts levels of *VrMybA1* and *VrMYCS-1* were influenced by the accumulation of anthocyanins in the berry skins and cell lines, implying that they play a vital role in anthocyanin accumulation. Therefore, *VrMybA1* and *VrMYCS-1* could be considered as potential targets in genetic engineering for producing transgenic plants with improved anthocyanin accumulation. To gain greater insight into the functions of *VrMybA1* and *VrMYCS-1* in anthocyanin accumulation in muscadine grapes, further research will focus on the analysis of *VrMybA1* and *VrMYCS-1* in transgenic *V. rotundifolia* plants and cell lines.
